# Google trends as a tool for evaluating public interest in total knee arthroplasty and total hip arthroplasty

**Published:** 2021-07-16

**Authors:** Samuel A. Cohen, Landon E. Cohen, Jonathan D. Tijerina, Gabriel Bouz, Rachel Lefebvre, Milan Stevanovic, Nathanael D. Heckmann

**Affiliations:** ^1^Stanford University School of Medicine 291 Campus Drive, Stanford, CA, 94305, USA; ^2^Keck School of Medicine, University of Southern California 1975 Zonal Avenue, Los Angeles, CA, 90033, USA; ^3^Bascom Palmer Eye Institute 900 NW 17^th^ St 2^nd^ floor, Miami, FL 33136, USA; ^4^Department of Orthopaedic Surgery, Keck School of Medicine, University of Southern California, 1975 Zonal Avenue, Los Angeles, CA, 90033, USA

**Keywords:** Google trends, Google, public interest, hip arthroplasty, knee arthroplasty

## Abstract

**Background and Aims:**

There are approximately 1 million total knee arthroplasty (TKA) and total hip arthroplasty (THA) procedures performed annually in the United States. With this number projected to increase, it is vital for orthopedic surgeons and health-care systems to properly anticipate healthcare utilization related to TKA and THA. Google Trends (GT) is a free, open source tool that provides customizable analysis of search terms entered into the Google search engine. We aim to explore the relationship between public interest in TKA and THA as determined by GT data and volume of TKA and THA procedures.

**Methods:**

GT data were compiled for ten search terms related to TKA and ten search terms related to THA from January 2009 to December 2017. Annual case volumes for TKA/THA procedures were obtained from the Healthcare Cost and Utilization Project National Inpatient Sample from 2009 to 2017. Trend analysis was performed using univariate linear regression of GT data and TKA/THA case volumes.

**Results:**

There was a statistically significant positive correlation between GT data and procedure volume for 14 of the 20 search terms studied. Seven TKA-related search terms with a positive correlation to procedure volumes include “total knee replacement,” “knee replacement,” “knee osteoarthritis,” “knee ache,” “knee swelling,” “knee stiffness,” and “chronic knee pain.” Seven THA-related search terms with a positive correlation to procedure volumes include “hip arthroplasty,” “total hip replacement,” “hip replacement,” “hip osteoarthritis,” “hip ache,” “hip swelling,” and “chronic hip pain.”

**Conclusion:**

GT may provide a high utility as a convenient and informative data set for orthopedic surgeons to analyze public interest in TKA and THA procedures. The data provided by GT have the potential to provide real-time, actionable information that may help surgeons and health systems to characterize public interest in TKA and THA and to best identify and address patient needs.

**Relevance for Patients:**

The GT tool can be used to measure public interest in TKA/THA, which can inform physician expectations for the patient encounter and lead to the creation of decision aids that better inform the public about the risks and benefits of TKA/THA.

## 1. Introduction

Total knee arthroplasty (TKA) and total hip arthroplasty (THA) are two of the most common orthopedic procedures, with more than 1 million TKA and THA surgeries performed each year in the United States [1]. An aging population is expected to significantly increase the number of TKA and THA procedures performed in the coming years, as the average age of TKA and THA recipients is 67 and 65 years, respectively [2,3]. In fact, TKA and THA procedures are expected to increase by 401% and 284% by 2040 when compared with 2012 case volumes [1]. An increase in TKA and THA volume may have significant economic consequences related to healthcare resource utilization, as patients undergoing TKA and THA often present with increased rates of age-related comorbidities which are associated with higher costs [4]. As the number of TKA and THA procedures continues to rise, it is vital for orthopedic surgeons and health-care systems to properly anticipate health-care utilization related to TKA and THA to inform resource allocation and staffing decisions that optimize patient outcomes.

There have been many recent attempts to monitor and characterize public interest in surgical procedures to better forecast surgical case volumes [5,6]. However, the majority of these forecasting techniques utilize complex mathematical models and are not intuitive to the typical surgeon. There has been limited research on the use of publicly available internet search traffic data to help forecast case volumes for orthopedic procedures. Google Trends (GT) is a free, open source tool that provides customizable analysis of search term volumes entered into the Google search engine [7]. Recent estimates indicate that the Google search engine receives more than 70,000 health-related searches per minute, which account for nearly 7% of all Google searches [8]. The GT tool has been used in a wide array of studies to track public interest in health topics ranging from interest in elective procedures to interest in cancer screening [9,10]. Previously, GT data describing public interest in several different types of surgical and non-surgical procedures have been shown to be correlated with case volumes [11-14]. Furthermore, GT has been used in previous orthopedic studies to examine seasonal trends in knee pain as well as to track public interest in stem cell injections and platelet-rich plasma for osteoarthritis of the hip and knee [15-17]. However, to date, there is a paucity of research examining the relationship between GT data and volumes of orthopedic procedures such as TKA and THA.

The purpose of our study is to explore the relationship between public interest in TKA and THA as determined by GT data and the volume of TKA and THA procedures performed in the United States. We also describe important temporal and seasonal patterns in public interest related to TKA and THA in the United States, providing insight that may help inform important staffing and resource allocation decisions that maximize productivity and improve patient outcomes.

## 2. Methods

### 2.1. GT analysis

GT analysis for a given search term can be filtered by time period and geographic location. After a search term is entered into GT and the appropriate temporal and geographic constraints specified, GT generates visuals that reflect the volume of a given search term relative to its peak popularity within the defined time period, which is assigned a value of 100. The data are presented as relative search volume (RSV), which is computed as the percentage of searches of a term in a location during a specific time period. An RSV value of 100 indicates the largest ratio between searches for a specific topic and the total amount of Google queries. A value of 0 indicates that at the specified time point, the proportion of queries for the search term was <1% of its peak RSV (RSV 100) [7].

Search terms related to TKA and THA were chosen using the “related queries” feature of GT. Databases of search volumes over time were collected for 20 search terms that were divided into four subgroups. Two subgroups were devoted to each procedure, with one subgroup focusing on different variations of the name of the procedure itself and one subgroup focusing on common symptoms that may indicate the given procedure is necessary. Search terms in the TKA procedure category were as follows: “total knee arthroplasty,” “knee arthroplasty,” “total knee replacement,” “knee replacement,” and “total knee replacement surgery.” Search terms in the TKA symptom category were as follows: “knee osteoarthritis,” “knee ache,” “knee swelling,” “knee stiffness,” and “chronic knee pain.” Search terms in the THA procedure category were as follows: “total hip arthroplasty,” “hip arthroplasty,” “total hip replacement,” “hip replacement,” and “total hip replacement surgery.” Search terms in the THA symptom category were as follows: “hip osteoarthritis,” “hip ache,” “hip swelling,” “hip stiffness,” and “chronic hip pain.” Colloquial terms as well as technical terms related to both TKA and THA were included in our analyses. We used GT’s customizable filters to include results for searches within the United States from January 2009 to December 2017 for all selected terms based on availability of case volumes for the years included in the study.

#### 2.1.1. Case volumes

Annual case volumes for both TKA and THA were obtained from the Healthcare Cost and Utilization Project (HCUP) National Inpatient Sample (NIS) annual reports from 2009 to 2017 using proper International Classification of Diseases, Ninth or Tenth (ICD9/10) Revision codes (Appendix A).

### 2.2. Statistical analysis

Univariate linear regression was used to evaluate the correlation between average annual GT data and annual volume of TKA and THA procedures, mimicking the protocol of other GT studies that examined the association between Google search volumes and case volumes [11-13,18]. We used the Bonferroni correction (0.05/10) to define statistical significance at *P*<0.0050 because of the increased risk of a type 1 error when performing multiple linear regressions. In addition, seasonal trends for all search terms with a statistically significant correlation to TKA and THA case volumes were observed using a month-by-month analysis of variation from yearly GT means. All statistical and trend analyses were performed using Microsoft Excel Version 15.21.1 and SPSS Version 26.0.0.1.

## 3. Results

### 3.1. Correlation of GT search volumes and TKA procedures in the United States

There was an average of approximately 671,000 annual TKA procedures from 2009 to 2017. Univariate linear regression of annual GT data from 2009 to 2017 compared with annual HCUP NIS TKA case volumes demonstrated statistically significant positive correlations for several terms. The search terms with a statistically significant positive correlation to TKA case volumes include “total knee replacement” (*P*=0.0010, R^2^=0.876), “knee replacement” (*P*<0.001, R^2^=0.982), “knee osteoarthritis” (*P*<0.001, R^2^=0.966), “knee ache” (*P*=0.0021, R^2^=0.816), “knee swelling” (*P*=0.0022, R^2^=0.813), “knee stiffness” (*P*<0.001, R^2^=0.947), and “chronic knee pain” (*P*=0.0014, R^2^=0.838). No significant correlation between GT search data and TKA case volumes was detected for the search terms “total knee arthroplasty,” “knee arthroplasty,” and “total knee replacement surgery” (Table 1).

**Table 1:** Relationship of Google trends search volumes and total knee arthroplasty case volumes

Google trends search term	Coefficient (95% CI)	*P* value	R^2^
Total knee arthroplasty	11350.0 (4485.1, 18215.0)	0.0068	0.732
Knee arthroplasty	7905.0 (2341.8, 13468.2)	0.0132	0.668
Total knee replacement	5042.0 (−3144.3, 6939.7)	0.0010	0.876
Knee replacement	5199.1 (4505.3, 5892.8)	<0.001	0.982
Total knee replacement surgery	4212.0 (−0.110, 1613..0)	0.0510	0.499
Knee osteoarthritis	4277.4 (3478.2, 5076.6)	<0.0010	0.966
Knee ache	4156.9 (2187.2, 6126.6)	0.0021	0.816
Knee swelling	−3874.1 (2018.3, 5730.0)	0.0022	0.813
Knee stiffness	6154.2 (4700.2, 7608.1)	<0.001	0.947
Chronic knee pain	5497.9 (−3079.8, 7916.0)	0.0014	0.838

### 3.2. Correlation of GT search volumes and THA procedures in the United States

There was an average of approximately 348,000 annual THA procedures from 2009 to 2017. Univariate linear regression of annual GT data from 2009 to 2017 compared with annual HCUP NIS THA case volumes demonstrated statistically significant positive correlations for several terms. The search terms with a statistically significant positive correlation to THA case volumes include “hip arthroplasty” (*P*<0.001, R^2^=0.939), “total hip replacement” (*P*<0.001, R^2^=0.919), “hip replacement” (*P*<0.001, R^2^=0.960), “hip osteoarthritis” (*P*<0.001, R^2^=0.912), “hip ache” (*P*<0.001, R^2^=0.951), “hip swelling” (*P*<0.001, R^2^=0.913), and “chronic hip pain” (*P*<0.001, R^2^=0.900). No significant correlation between GT search data and THA case volumes was detected for the search terms “total hip arthroplasty,” “total hip replacement surgery,” and “hip stiffness” (Table 2).

**Table 2:** Relationship of Google trends search volumes and total hip arthroplasty case volumes

Google trends search term	Coefficient (95% CI)	*P* value	R^2^
Total hip arthroplasty	6079.2 (579.8, 11578.6)	0.0353	0.549
Hip arthroplasty	10750.7 (8015.4, 13485.9)	<0.001	0.939
Total hip replacement	7282.0 (5115.3, 9448.5)	<0.001	0.919
Hip replacement	5955.9 (4742.5,7169.2)	<0.001	0.960
Total hip replacement surgery	3570.5 (−4157.6, 11298.7)	0.3014	0.176
Hip osteoarthritis	4400.8 (3032.3, 5769.3)	<0.001	0.912
Hip ache	5535.3 (4279.1, 6791.5)	<0.001	0.951
Hip swelling	4633.9 (3202.3, 6064.4)	<0.001	0.913
Hip stiffness	6229.9 (−866.6, 13326.4)	0.0753	0.435
Chronic hip pain	6115.1 (4075.2, 8115.1)	<0.001	0.900

### 3.3. Trends in overall interest in search terms

Among the TKA procedure search terms with a statistically significant positive correlation to case volumes, search traffic was highest for the term “knee replacement” followed by “total knee replacement” (Figure 1A). Among the TKA symptom search terms with a statistically significant positive correlation to case volumes, search traffic was highest for the term “knee swelling,” followed by “knee osteoarthritis,” “knee stiffness,” “knee ache,” and “chronic knee pain” (Figure 1B).

**Figure 1A:**
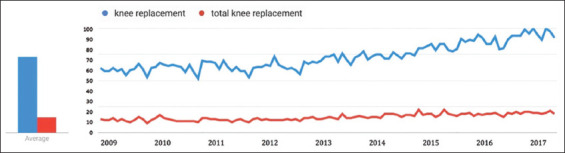
Temporal trends in public interest in total knee arthroplasty procedure search terms. Trends in the average monthly search volumes for total knee arthroplasty procedure search terms in the US from January 2009 to December 2017. Level of interest is expressed on a scale of 0% to 100%, with 100% interest reflecting the month with the highest search volume. (Blue = “knee replacement”; Red = “total knee replacement” [Data source: Google Trends (www.google.com/trends)]).

**Figure 1B:**
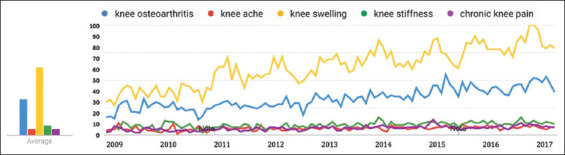
Temporal trends in public interest in total knee arthroplasty symptom search terms. Trends in the average monthly search volumes for total knee arthroplasty symptom search terms in the US from January 2009 to December 2017. Level of interest is expressed on a scale of 0% to 100%, with 100% interest reflecting the month with the highest search volume. (Blue = “knee osteoarthritis”; Red = “knee ache”; Yellow = “knee swelling”; Green = “knee stiffness”; Purple = “chronic knee pain”) [Data source: Google Trends (www.google.com/trends)]).

Among the THA procedure search terms with a statistically significant positive correlation to case volumes, search traffic was highest for the term “hip replacement,” followed by “total hip replacement,” and “hip arthroplasty” (Figure 1C). Among the THA symptom search terms with a statistically significant positive correlation to THA case volumes, search traffic was highest for the term “hip osteoarthritis,” followed by “hip swelling,” “hip ache,” and “chronic hip pain” (Figure 1D).

**Figure 1C:**
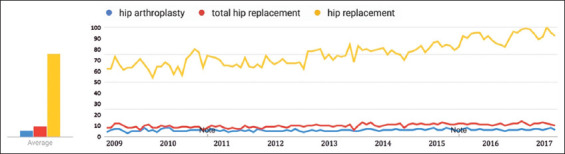
Temporal trends in public interest in total hip arthroplasty procedure search terms. Trends in the average monthly search volumes for total hip arthroplasty procedure search terms in the US from January 2009 to December 2017. Level of interest is expressed on a scale of 0% to 100%, with 100% interest reflecting the month with the highest search volume. (Blue = “hip arthroplasty”; Red = “total hip replacement”; Yellow = “hip replacement” [Data source: Google Trends (www.google.com/trends)]).

**Figure 1D:**
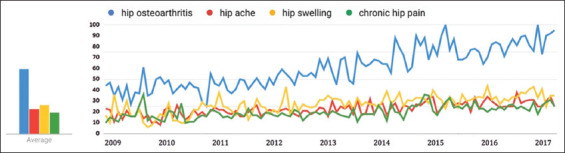
Temporal trends in public interest in total hip arthroplasty symptom search terms. Trends in the average monthly search volumes for total hip arthroplasty symptom search terms in the US from January 2009 to December 2017. Level of interest is expressed on a scale of 0% to 100%, with 100% interest reflecting the month with the highest search volume. (Blue = “hip osteoarthritis”; Red = “hip ache”; Yellow = “hip swelling”; Green = “chronic hip pain”) [Data source: Google Trends (www.google.com/trends)]).

### 3.4. Seasonal trends

Search terms within each of the four aforementioned categories with a statistically significant positive correlation to TKA/THA case volumes were grouped together for seasonal analyses. Group 1 (TKA Procedure Terms) consisted of the search terms “total knee replacement,” and “knee replacement.” Group 2 (TKA Symptom Terms) consisted of the search terms “knee osteoarthritis,” “knee ache,” “knee swelling,” “knee stiffness,” and “chronic knee pain.” Group 3 (THA Procedure Terms) consisted of the search terms “hip arthroplasty,” “total hip replacement,” and “hip replacement.” Group 4 (THA Symptom Terms) consisted of the search terms “hip osteoarthritis,” “hip ache,” “hip swelling,” and “chronic hip pain.” Within each group, average monthly RSV was calculated for each month from January 2009 to December 2017.

Peak interest in TKA Procedure search terms (Group 1) was observed in the months of October (+3.5%), February (+3.3%), and June (+2.1%). Minimal interest in TKA procedure search terms (Group 1) was observed in the months of January (−3.5%), December (−3.2%), and August (−3.0%), with no clear seasonal patterns (Figure 2A and Table 3).

**Figure 2A:**
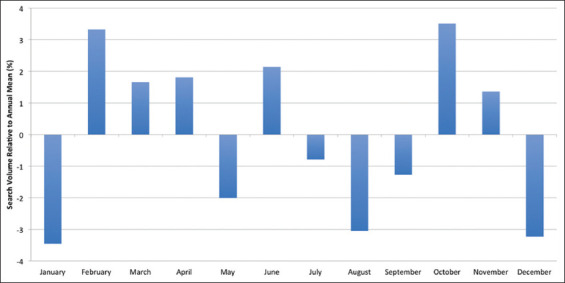
Monthly interest in total knee arthroplasty procedure search terms. Peak public interest in total knee arthroplasty procedure search terms was in the months of October, February, and June. Least public interest in total knee arthroplasty procedure search terms was in the months of January, December, and August.

**Table 3:** Seasonal trends in Google trends search volumes related to total knee arthroplasty and total hip arthroplasty

Google trends search category	Mean search volume relative to peak (%)			

	Winter	Spring	Summer	Fall
Total knee arthroplasty procedure-related	66.4	68.0	67.0	68.8
Total knee arthroplasty symptom-related	52.8	59.2	63.4	58.9
Total hip arthroplasty procedure-related	67.7	73.9	70.5	74.6
Total hip arthroplasty symptom-related	51.4	52.1	53.0	55.7

Peak interest in TKA Symptom search terms (Group 2) was observed in the months of June (+8.5%), July (3.4%), and August (+2.6%). Minimal interest in TKA symptom search terms (Group 2) was observed in the months of February (−7.8%), December (−6.1%), and January (−3.3%), with a clear pattern of decreased search traffic in the winter months of December-February and increased search traffic in the summer months of June-August (Figure 2B and Table 3).

**Figure 2B:**
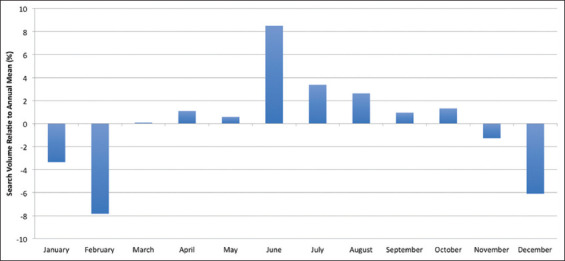
Monthly interest in total knee arthroplasty symptom search terms. Peak public interest in total knee arthroplasty symptom search terms was in the months of June, July, and August. Least public interest in total knee arthroplasty symptom search terms was in the months of February, December, and January.

Peak interest in THA Procedure terms (Group 3) was observed in the months of October (+7.2%), April (+5.8%), and November (+2.7%). Minimal interest in THA procedure search terms (Group 3) was observed in the months of December (−8.0%), January (−5.6%), and August (−3.1%), with a less pronounced pattern of decreased interest in the winter months of December-February when compared with the rest of the year (Figure 2C and Table 3).

**Figure 2C:**
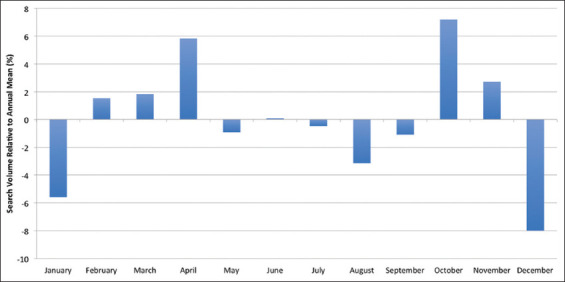
Monthly interest in total hip arthroplasty procedure search terms. Peak public interest in total hip arthroplasty procedure search terms was in the months of October, April, and November. Least public interest in total hip arthroplasty procedure search terms was in the months of December, January, and August.

Peak interest in THA Symptom terms (Group 4) was observed in the months of October (+6.6%), November (3.1%), and July (+1.9%). Minimal interest in THA symptom search terms (Group 4) was observed in the months of January (−3.3%), June (−2.2%), and September (−2.0%), with no clear seasonal patterns (Figure 2D and Table 3).

**Figure 2D:**
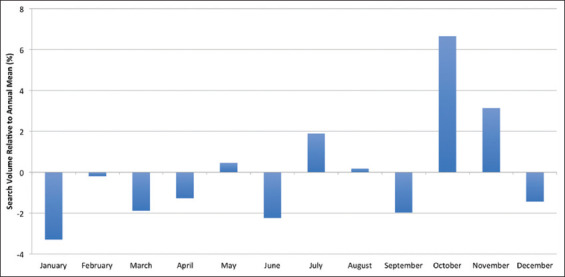
Monthly interest in total hip arthroplasty symptom search terms. Peak public interest in total hip arthroplasty symptom search terms was in the months of October, November, and July. Least public interest in total hip arthroplasty symptom search terms was in the months of January, June, and September.

## 4. Discussion

Our study demonstrates the value of GT in monitoring and characterizing patient interest in TKA and THA procedures over time. Univariate linear regression demonstrated statistically significant positive correlations between GT data and TKA/THA procedure volumes for 14 of the 20 search terms studied. Our results add to the growing body of evidence suggesting that GT is a tool that can be used to characterize trends in case volumes for surgical procedures [11-13,18,19]. While many GT studies have focused on the relationship between GT data and case volumes, the majority of the procedures previously analyzed were cosmetic or dermatological in nature [9,10]. Our study is the first that we know of to examine the relationship between GT data and case volumes for TKA and THA. In addition, increased public interest in TKA/THA over time, as reflected by greater GT search volumes, indicates the need for the creation of decision aids that discuss the risks and benefits of TKA/THA to inform an increasingly curious public about the respective procedures.

The previous studies examining the association between GT data and surgical case volumes have discussed the importance of careful section of search terms [11,19]. Our results indicate that for TKA and THA the large majority of search terms generated using the “related queries” feature of GT were positively correlated with case volumes. However, similar to the previous studies [11,12], colloquial rather than technical names associated with TKA and THA resulted were more strongly associated with case volumes. In addition, while many other GT studies have examined the correlation between Google search volumes for the name of a given procedure (i.e., ”rhinoplasty,” “otoplasty,” and “rhytidectomy”) and actual case volumes, our study also examines the association between search terms describing popular symptoms and/or precursors to TKA/THA and case volumes.

The ability to use GT to monitor and characterize public interest in both TKA and THA has important consequences for orthopedic surgeons and healthcare systems alike. A review of medical claims data from 2010 to 2017 by the Blue Cross Blue Shield Association (BCBSA) discovered that knee replacements and hip replacements increased by 17% and 33%, respectively, from 2010 to 2017 [20]. As such, the ability to anticipate future TKA and THA volume can provide orthopedic surgeons and health-care systems with valuable information that can inform resource allocation decisions that impact efficiency and productivity. McIntosh *et al*. [21] recently explored many different factors that impact operating room (OR) productivity. Their results indicate that the most important predictor of OR productivity was the refinement of operating room allocations such as service-specific staffing decisions that occur 2–3 months before the day of surgery [21]. GT data can provide orthopedic surgeons and health-care systems with real-time information about public interest in TKA and THA-related search terms at the state, county, and even city level that can inform staffing decisions several months in advance, thereby optimizing productivity, and efficiency in the OR. GT may be particularly useful for resource-limited hospitals in many parts of the country, where OR time is at a premium [22].

In addition to staffing decisions, trends in GT data can also be used to optimize OR scheduling. Operating rooms are typically a hospital’s greatest revenue source [23]. As a result, there has been extensive research examining various machine learning models that can be used to improve OR scheduling and therefore increase efficiency and decreases costs [24-26]. These machine learning models, however, are often complex and therefore inaccessible to a non-technologically advanced orthopedic surgeon. Our data suggest that GT can be utilized as a powerful, intuitive supplement to more complex machine learning models in order to characterize and anticipate public interest in TKA and THA. GT data tracking public interest in a given procedure such as TKA/THA can be combined with other “big data” that provides additional demographic information to gain a clearer understanding of who and what is driving various healthcare trends, which can result in more informed procedure forecasting and more efficient scheduling. GT provides actionable data that can be used by orthopaedic surgeons and health-care systems to match the supply of OR time with demand for TKA and THA procedures as temporal, seasonal, and geographic trends dictate.

The ability to characterize public interest in TKA and THA will be critical to orthopedic surgeons and health-care system that wish to anticipate healthcare resource utilization by patients who endure TKA and THA. The previous literature discusses the increased utilization of health-care resources by patients with osteoarthritis compared to individuals without osteoarthritis [27-30]. Increased health-care utilization by patients with osteoarthritis is largely attributable to the great economic burden associated with late-stage osteoarthritis procedures such as TKA and THA [27]. In addition, the number of both TKA and THA procedures is only expected to increase in the upcoming years as a result of an aging and increasingly obese American population, with one report warning that even the most conservative projections predict a 143% increase in TKA by 2050 compared with 2012 [31-33]. A lack of clarity about TKA inpatient/outpatient status may further compound problems associated with increasing demand for TKA. On January 1, 2018, TKA was removed from the Centers for Medicare and Medicaid Services (CMS) Inpatient-Only list [34]. A survey administered by the American Association of Hip and Knee Surgeons reveals that as a result of the CMS decision, approximately 60% of survey respondents reported that their hospitals had required all Medicare TKAs to be scheduled as outpatient procedures. In addition, 76.1% of respondents reported the issue was an “administrative burden” [34]. The CMS decision has financial consequences not only for health-care systems but also for patients, as nearly 30% of TKA surgeons reported that their patients were subject to increased costs as a result of the TKA being billed as an outpatient rather than inpatient procedure [34]. These increased costs may compound the already disproportionate economic burden on patients who live with hip and knee osteoarthritis and subsequently undergo TKA and THA [35]. The uncertainty regarding the billing status of TKA and other surgical procedures that may continue as healthcare policy evolves is another reason why the ability to anticipate public interest in procedures such as TKA and THA is so important. Our results indicate that GT tool can serve as a supplement to more complex predictive models and provide insight about staffing and resource allocation decisions that optimize outcomes for both the patient and the health-care provider alike.

The power of the GT tool to characterize public interest in medical conditions and procedures in real-time has many clinical implications. For one, GT may help to track public interest in more controversial procedures/therapies that have not yet been proven effective, which can lead to policy changes or recommendations. For example, the previous reports indicate an increase in public interest for both stem cells and platelet-rich plasma to treat hip/knee osteoarthritis, despite the fact that limited evidence exists for the use of either therapy [16,17]. When GT data demonstrate that public interest in an unproven therapy is increasing, it is vital that medical personnel and organizations make a concerted effort to release guidelines that provide the public with trusted information about the therapy, which is often difficult to find on the internet -- where medical information is often misleading or false [36,37]. Second, unlike many current medical research databases that provide medical utilization information about patients nearly 2 years after insurance claims are submitted, GT can be used to measure abrupt, real-time changes in public interest in medical conditions or procedures, which can help to gauge the public’s response to changing health-care policy and to better inform forecasting models.

In addition to informing resource allocation decisions in the present, seasonal GT data may also provide answers to weather-related joint pain questions in the future. Our results indicate that although peak GT interest for TKA and THA-related search terms varied, seasonal trends indicate decreased search volumes for all TKA and THA-related search terms in the winter months of December-February compared with the March-November months in the spring, summer, and fall seasons (Table 3). These results align with a recent study that reported increased Google searches for “knee swelling” and “knee pain” in the warmer months when compared with colder months [15]. There have been many attempts to determine the association between weather conditions and osteoarthritis pain [38-40]. One recent report revealed that in a study of 222 people with hip osteoarthritis, pain, and stiffness worsened with increased barometric pressure and humidity [38]. Another large study found no association between weather conditions and joint pain [41]. While there is no scientific consensus on the relationship between weather and joint pain, future study can employ GT data to discover a possible link between weather conditions and self-reported hip and knee pain. GT’s customizable analyses can provide real-time information about Google searches related to joint pain that can be matched with real-time weather conditions to determine if such a link exists. This information may help orthopedic surgeons to better identify and anticipate knee and hip complaints that ultimately require TKA and THA, respectively.

There are several limitations to our study. First, GT provides RSV rather than raw values of Google searches for search terms that are entered into the Google search engine. In addition, Google provides very limited demographic data about the users whose search traffic data are represented in this study. As a result, it is difficult to discern how many unique users are being reflected in the GT data. More specific demographic information about the age of users searching for information about TKA and THA would be beneficial to future GT study. Furthermore, our analysis is limited by the fact that GT only provides data about search trends for search terms with sufficient search traffic to report. There are other search terms related to TKA and THA that did not populate in the “related queries” feature of GT due to insufficient search traffic and that may help explain some of the trends observed in our study. Finally, we must be careful not to confuse correlation with causation when examining the relationship between GT search volumes and actual procedure volumes for TKA and THA. Previously, many factors including the overall health of the economy and insurance status have been shown to influence whether or not a patient proceeds with elective surgery [42,43]. The use of the internet to seek more information about TKA and THA as well as common hip and knee symptoms is just one of many factors that may contribute to whether or not an individual ultimately has TKA or THA. Despite these limitations, we believe that our results suggest that GT represents a convenient, intuitive tool that can be used a supplement to more traditional procedure forecasting models by orthopedic surgeons in order to characterize public demand for TKA and THA.

## 5. Conclusions

GT is a free, open-source tool that has previously been shown to be useful in characterizing case volumes for many elective surgical procedures; however, there has been little study about the association between GT data and case volumes for orthopedic conditions such as TKA and THA. Our study demonstrates that search volumes associated with many GT search terms related to TKA and THA exhibit statistically significant positive correlations with annual case volumes. This information may provide valuable insight to orthopedic surgeons and surgical centers that hope to characterize public interest in TKA and THA procedures for both staffing and resource allocation purposes.

### Conflict of Interest

The authors declare no conflicts of interest.

## Appendix

**Appendix A:** International Classification of Diseases (ICD) Ninth and Tenth Revision Codes for total knee arthroplasty and total hip arthroplasty

Total knee arthroplasty	ICD9: 81.54
	ICD10: 0SRC07Z; 0SRC0J9; 0SRC0JA; 0SRC0JZ; 0SRC0KZ; 0SRC0L9; 0SRC0LA; 0SRC0LZ; 0SRT07Z; 0SRT0J9; 0SRT0JA; 0SRT0JZ; 0SRT0KZ; 0SRV07Z; 0SRV0J9; 0SRV0JA; 0SRV0JZ; 0SRV0KZ; 0SRD07Z; 0SRD0J9; 0SRD0JA; 0SRD0JZ; 0SRD0KZ; 0SRD0L9; 0SRD0LA; 0SRD0LZ ; 0SRU07Z; 0SRU0J9; 0SRU0JA; 0SRU0JZ; 0SRU0KZ; 0SRW07Z; 0SRW0J9; 0SRW0JA; 0SRW0JZ; 0SRW0KZ
Total hip arthroplasty	ICD9: 81.51
	ICD10: 0SR9019; 0SR901A; 0SR901Z; 0SR9029; 0SR902A; 0SR902Z; 0SR9039; 0SR903A; 0SR903Z; 0SR9049; 0SR904A; 0SR904Z, 0SR907Z, 0SR90J9, 0SR90JA, 0SR90JZ, 0SR90KZ, 0SRA009, 0SRA00A, 0SRA00Z, 0SRA019, 0SRA01A, 0SRA01Z, 0SRA039, 0SRA03A, 0SRA03Z, 0SRA07Z, 0SRA0J9, 0SRA0JA, 0SRA0JZ, 0SRA0KZ, 0SRR019, 0SRR01A, 0SRR01Z, 0SRR039, 0SRR03A, 0SRR03Z, 0SRR07Z, 0SRR0J9,
	0SRR0JA, 0SRR0JZ, 0SRR0KZ, 0SRB019, 0SRB01A, 0SRB01Z, 0SRB029, 0SRB02A, 0SRB02Z, 0SRB039, 0SRB03A, 0SRB03Z, 0SRB049, 0SRB04A, 0SRB04Z, 0SRB07Z, 0SRB0J9, 0SRB0JA, 0SRB0JZ, 0SRB0KZ, 0SRE009, 0SRE00A, 0SRE00Z, 0SRE019, 0SRE01A, 0SRE01Z, 0SRE039, 0SRE03A, 0SRE03Z, 0SRE07Z, 0SRE0J9, 0SRE0JA, 0SRE0JZ, 0SRE0KZ, 0SRS019, 0SRS01A, 0SRS01Z, 0SRS039, 0SRS03A, 0SRS03Z, 0SRS07Z, 0SRS0J9, 0SRS0JA, 0SRS0JZ, 0SRS0KZ
